# The different clinical guideline standards in Brazil: High cost treatment diseases versus poverty-related diseases

**DOI:** 10.1371/journal.pone.0204723

**Published:** 2018-10-17

**Authors:** Rafael Santos Santana, Evandro de Oliveira Lupatini, Fernando Zanghelini, Ricardo de March Ronsoni, Norberto Rech, Silvana Nair Leite

**Affiliations:** 1 Department of Pharmacy, University of Brasília, Brasília, Federal District, Brazil; 2 Postgraduate Program in Collective Health, University of Brasília, Brasília, Federal District, Brazil; 3 Postgraduate Program in Therapeutic Innovation, Federal University of Pernambuco, Recife, Pernambuco, Brazil; 4 Department of Pharmaceutical Assistance, Ministry of Health, Brasília, Federal District, Brazil; 5 Department of Pharmaceutical Sciences, Federal University of Santa Catarina, Florianópolis, Santa Catarina, Brazil; Flinders University, AUSTRALIA

## Abstract

Each year, evidence-based clinical guidelines gain more space in the health professionals’ practice and in services organization. Due to the scarcity of scientific publications focused on diseases of poverty, the development of well-founded clinical guidelines becomes more and more important. In view of that, this paper aims to evaluate the quality of Brazilian guidelines for those diseases. The AGREE II method was used to evaluate 16 guidelines for poverty-related diseases (PRD) and 16 guidelines for global diseases whose treatment require high-cost technologies (HCD), with the ultimate aim of comparing the results. It was found that, in general, the guideline development quality standard is higher for the HCD guidelines than for the PRD guidelines, with emphasis on the "rigour of development" (48% and 7%) and "editorial independence" (43% and 1%) domains, respectively, which had the greatest discrepancies. The HCD guidelines showed results close to or above international averages, whereas the PRD guidelines showed lower results in the 6 domains evaluated. It can be concluded that clinical protocol development priorities need some redirecting in order to qualify the guidelines that define the healthcare organization and the care of vulnerable populations.

## Introduction

The advancement of evidence-based health care has positively impacted patient care, improving diagnostic accuracy, guiding healthcare professionals on using the most effective therapy, and reducing patient exposure to ineffective or even harmful interventions [1].

In view of the great advance of scientific studies in the health area, one can state that access to the best available evidence is a right of the health professional and the patient, target of the health intervention. Well-developed clinical guidelines have the power to mediate different interests in health care policy, best practice, government funding, local contexts, and patient choice [2].

Yet, the great number of clinical guidelines continuously produced without an acceptable quality standard that supports their recommendations leads to discrediting of such a fundamental mechanism of health care qualification. Several guidelines developed for the care of the same disease and produced with different methods and objectives can generate unnecessary competition and a complex system of conflicting practices and interventions [3].

In Brazil, since 2000 there has been a movement for clinical guideline qualification using structured methods for the development and publication of the so-called “Clinical Protocols and Therapeutic Guidelines (PCDTs)”. Early versions of those documents include a small number of clinical conditions for which the cost of treatment, especially medicines, was considerably high.

After the enactment of Law No. 12,401 and Decree No. 7,508, both in 2011 (on therapeutic care and technology incorporation in the Brazilian health system), the PCDTs began to gain institutional legitimacy and greater coverage, becoming a reference document for disease or condition diagnosis, pharmacological and non-pharmacological treatment, patient follow-up and verification of therapeutic results [4]. Thus, in the context of the Brazilian Unified Health System (SUS, the national public health system), the PCDTs are referred to as Clinical Practice Guidelines, sometimes shortened as Clinical Guidelines.

The PDCTs are applied, also, on regulating and controlling the free access to high-cost treatment. High-cost medicines is an expression without a uniform definition but adopted by the Pan American Health Organization and partner countries to define therapies that are the major burden for the financing of the health system and / or high household expenditure. It is characterized generally by a greater interest of the pharmaceutical industry and consequently more number of launches / innovations and patents. These medicines are particularly applied for the so called “global diseases”, those that did not have the socioeconomic vulnerability bias as a fundamental determinant [5,6,7,8]. In Brazil, those diseases are covered by a program called Specialized Component of Pharmaceutical Assistance [4].

On the other hand, according to the conceptual propositions of the Médecins Sans Frontières (MSF, or Doctors Without Borders) and the World Health Organization (WHO), tropical, neglected or related to poverty diseases have opposite characteristics, with less interest of the pharmaceutical industry and consequently smaller number of medicines available, innovations and patents. They are often characterized by requesting older and less expensive drugs for the health system. In Brazil, these drugs are mostly produced by public laboratories and covered by the public program called Strategic Conditions Component of the Pharmaceutical Assistance [5, 6]. In this country, poverty-related diseases are particularly important health issues. They are the result of poor living conditions, insufficient access to adequate and healthy food, clean drinking water, medical care and education [5,6,7,8]. Some infectious diseases such HIV, considered related to poverty globally, in Brazil is not classified as neglected or poverty related. Particular healthcare facilities and guidelines are available and accessible.

Most of the so-called poverty-related and neglected diseases and conditions, however, are still addressed by the SUS in the form of different documents such as guides, manuals and handbooks formulated in diverse methodological ways rather than in a standardized way in line with the PCDTs’ structures.

In this sense, this paper aims to evaluate the methodological quality of the documents published by the Brazilian Ministry of Health addressing care guidelines for the population affected by poverty-related diseases, in an attempt to verify their compliance with evidence-based health standards and consequently the needs of health caregivers and patients.

## Methods

### Identification and selection of clinical guidelines

The study included only clinical guidelines published by the Brazilian Ministry of Health for the diseases or conditions covered by the Specialized Component of Pharmaceutical Assistance and the Strategic Conditions Component of the Pharmaceutical Assistance. Documents issued by states, municipalities or other health institutions were not included in the analysis. Likewise, posters, flyers or like documents that did not fit the structure of care guides or protocols were not considered, even if they contained therapeutic conduct guidelines.

Given the variety of documents for the same disease or condition, the quality assessment included the most recently published ones and with the most detailed information on care (clinical condition, diagnostic and therapeutic conduct and the target population of the proposed interventions). At that moment (2016), sixteen documents had been published by the Ministry of Health about diseases or conditions classified as poverty-related, presenting treatment guidelines and covered by the Strategic Conditions Component of Pharmaceutical Assistance. As a comparison criterion with the global disease guidelines, the study sample included all the PCDTs issued by the CONITEC (National Committee for Health Technology Incorporation in the SUS) and published as Ministry of Health Ordinances in 2015, the immediate year before the study. Sixteen PCSTs were published in that year. The main characteristic of these PCDTs is their focus on the so-called 'high-cost diseases', included in the Specialized Component of Pharmaceutical Assistance (CEAF). The 32 guidelines selected for the study are described in Table 1:

**Table 1:** Clinical guidelines of the Brazilian Ministry of Health selected for the study.

Acronym	Selected guideline (translated from the Portuguese title)
PRD 01	Health Surveillance Guide (2014)
PRD 02	Guide to Infectious and Parasitic Diseases (2010)
PRD 03	Reference Guide to Epidemiologic Surveillance, Nutrition Assistance in Beriberi Cases (2012)
PRD 04	NutriSUS: evidence guidelines: food fortification strategies for infant nutrition with micronutrient (vitamins and minerals) powders (2015)
PRD 05	General Guidelines of the National Vitamin A Supplementation Program (2013)
PRD 06	National Iron Supplementation Program: General Guidelines (2013)
PRD 07	Dengue: Diagnosis and Clinical Management - Adults and Children (2016)
PRD 08	Sickle Cell Disease: Basic Guidelines for the Treatment (2012)
PRD 09	Schistosomiasis Mansoni Surveillance: Technical Guidelines (2014)
PRD 10	Protocol on Health Care and Response to the Occurrence of Microcephaly Related to Zika Virus Infection (2016)
PRD 11	Guide to Epidemiologic Surveillance and Elimination of Lymphatic Filariasis (2009)
PRD 12	Technical Operations Manual - Guidelines for surveillance, care and elimination of leprosy as a public health problem (2016)
PRD 13	Leptospirosis: Diagnosis and Clinical Management (2014)
PRD 14	PCDTs for Comprehensive Care for People with Sexually Transmitted Infections (2016)
PRD 15	Guide to Surveillance and Elimination of Trachoma as a Cause of Blindness (2014)
PRD 16	Prevention and treatment of injuries caused by sexual violence against women and girls (2012)
HCD 17	PCDT Rheumatoid Arthritis (2015)
HCD 18	PCDT Chronic Hepatitis C Virus Infection and Co-Infections (2015)
HCD 19	PCDT Breast Cancer (2015)
HCD 20	PCDT Reactive Arthritis (2015)
HCD 21	PCDT Head and Neck Cancer (2015)
HCD 22	PCDT Celiac Disease (2015)
HCD 23	PCDT Amyotrophic Lateral Sclerosis (2015)
HCD 24	PCDT Multiple Sclerosis (2015)
HCD 25	PCDT Hyperprolactinemia (2015)
HCD 26	PCDT Congenital Hypothyroidism (2015)
HCD 27	PCDT Hereditary Ictioses (2015)
HCD 28	PCDT Primary Adrenal Insufficiency (2015)
HCD 29	PCDT Myasthenia Gravis (2015)
HCD 30	PCDT Multiple Myeloma (2015)
HCD 31	PCDT Guillain-Barré Syndrome (2015)
HCD 32	PCDT Non-infectious Uveitis (2015)

PRD = Poverty-Related Diseases; HCD = High-Cost Diseases

() Last update

### Methodological quality assessment: The AGREE II instrument

The quality analysis of the selected clinical guidelines was performed using the AGREE–Appraisal of Guidelines for Research and Evaluation, an assessment instrument developed from analyses of over 100 guidelines selected and evaluated independently by more than 200 evaluators from different parts of the world. It is used by the WHO and various technology assessment agencies worldwide as a reference tool for quality assessment of clinical practice guidelines. Its last edition (AGREE II) has 23 key items organized within six quality domains [9,10].

Following the AGREE II instructions, the guidelines selected for this study were appraised by four independent experts with previous experience in clinical practice guideline assessment [11]. As commonly observed, similar studies have been using two or three appraisers; however the instrument recommends ideally four appraisers [9,11–13].

### Data extraction, management and analysis

Data were collected from the six quality domains described in the AGREE II Instrument: (i) scope and purpose; (ii) stakeholder involvement; (iii) rigour of development; (iv) clarity of presentation; (v) applicability, and (vi) editorial independence. The instrument provides a score sheet with ratings from 1 (strongly disagree) to 7 (strongly agree) for each of the 23 items. At the end, a quality score is calculated for each of the six AGREE II domains [11]. A percentage of suitability of each domain with values from 0% to 100% was also calculated using the score obtained by each evaluator and possible maximum score of the domain, following the guidelines of the AGREE II Instrument.

Although not suggested by the instrument, this study performed a concordance analysis among the appraisers, using the *kappa* test in order to avoid randomness or poor agreement. *Kappa* coefficients of moderate agreement (*kappa*> 0.4) were considered preferable for this type of study [12, 14].

For the concordance analysis, the appraisers decided jointly that scores 1 and 2 would be considered "low", scores between 3 and 5 would be "intermediate", and scores 6 and 7 "high". By evaluating the agreement between these categories, an initial *kappa* of 0.263 was obtained. Subsequently, a meeting was held with the appraisers to discuss the main dissenting opinions on the assessment criteria. The major divergences were remedied by re-reading and discussing together (all the appraisers) guidelines of the AGREE II Instrument [11]. After a new independent assessment, a final kappa of 0.598 was obtained, corresponding to a moderate agreement.

The non-parametric Mann-Whitney test was applied to compare the mean score difference of the guidelines in the six domains. The results were compared using program Statistical Package for the Social Sciences—SPSS (IBM, v. 20) with the level of significance set at 5%.

The instrument does not define a standard that indicates whether or not the guideline as a whole should be recommended. So, in order to make the assessment less subjective in that respect, it was agreed in this study that the domain (iii) “rigor of development” would be the main standard for the overall assessment of the guideline. Based on criteria suggested by other authors [9,10,12] the evaluators defined 50% as the minimum score for “rigor of development”, although some authors suggest a higher one. For a guideline to be considered as "recommended" it had to score higher than 50% in "rigor of development" and in two other domains; the guideline that scored between 30% and 50% in "rigor of development" and higher than 50% in two other domains was considered "recommended, with modifications"; and, lastly, the guideline that scored less than 30% in "rigor of development" was considered "not recommended".

## Results and discussion

The various types of guideline documents evince the dissent among their denominations: "primary care handbooks", "guides", "protocols", "general guidelines", "manuals", "technical guidelines", among other terms adopted by the Brazilian Ministry of Health. Indeed, there are some conceptual differences between those terms, but there also seems to be no consensus in the Brazilian literature about the standard designation of this type of document, which we have chosen here to call generically as a “clinical guideline”. However, the lack of standardized publications by the federal health agency may hinder the access to this information, both for researchers and target health professionals [9,10,12].

The diversity of healthcare recommendations for the same disease seems harmful insofar as they can generate conflicts concerning the suggested clinical conduct given the different guideline production methods used and the different results presented in the publications in question.

The AGREE II instrument, however, is sensitive to different types of guidelines as it assesses comprehensive and necessary issues for any type of therapeutic care guideline. The assessment results are expressed as quality scores (0 to 100) for each domain and each of the 32 guidelines validated in this study (Table 2).

**Table 2:** Quality scores of the guidelines assessed as per the AGREE II instruction.

Guideline	Scope and purpose	Stakeholder involvement	Rigour of development	Clarity of presentation	Applicability	Editorial independence
PRD 01	65%	10%	5%	51%	24%	4%
PRD 02	46%	24%	1%	43%	10%	4%
PRD 03	68%	26%	8%	54%	40%	4%
PRD 04	57%	24%	11%	51%	25%	2%
PRD 05	86%	31%	7%	53%	43%	0%
PRD 06	74%	18%	5%	60%	39%	0%
PRD 07	82%	7%	4%	58%	22%	0%
PRD 08	75%	7%	6%	65%	20%	0%
PRD 09	81%	40%	8%	71%	44%	0%
PRD 10	88%	35%	4%	65%	35%	0%
PRD 11	63%	26%	6%	54%	31%	0%
PRD 12	74%	31%	6%	67%	33%	0%
PRD 13	76%	35%	3%	75%	32%	0%
PRD 14	88%	58%	19%	79%	45%	0%
PRD 15	79%	21%	4%	57%	35%	0%
PRD 16	78%	24%	8%	68%	42%	0%
HCD 17	94%	40%	57%	93%	30%	46%
HCD 18	97%	40%	53%	90%	25%	40%
HCD 19	89%	39%	53%	75%	33%	27%
HCD 20	83%	42%	52%	65%	22%	46%
HCD 21	81%	43%	43%	61%	33%	27%
HCD 22	86%	44%	47%	58%	26%	46%
HCD 23	86%	44%	46%	75%	23%	46%
HCD 24	88%	44%	45%	76%	26%	46%
HCD 25	85%	44%	49%	74%	22%	46%
HCD 26	85%	44%	44%	63%	19%	46%
HCD 27	85%	44%	49%	63%	24%	46%
HCD 28	83%	42%	46%	64%	23%	46%
HCD 29	85%	44%	46%	72%	27%	46%
HCD 30	85%	44%	42%	61%	30%	46%
HCD 31	85%	44%	45%	61%	21%	46%
HCD 32	83%	43%	45%	69%	22%	46%

Table 3 displays the assessment results in a direct comparison between the two guideline groups evaluated (PRD x HCD), based on the ratings (1 to 7) obtained in each domain. There was a statistically significant difference between the mean scores contrasted.

**Table 3:** Mean score of each domain comparing the guideline groups of poverty-related diseases and high-cost diseases as per the AGREE II.

Quality domain	PRD	HCD	P Value
Scope and purpose	**5.4** ±0,69	**6.2** ±0,25	< 0.01
Stakeholder involvement	**2.6** ±0,78	**3.6** ±0,11	< 0.01
Rigour of development	**1.4** ±0,25	**3.9** ±0,25	< 0.01
Clarity of presentation	**4.6** ±0,59	**5.2** ±0,62	< 0.01
Applicability	**2.9** ±0,59	**2.5** ±0,27	< 0.05
Editorial independence	**1.1** ±0,10	**3.6** ±0,39	< 0.01

### Domain 1—scope and purpose

Fundamentally, this domain aims to analyze whether the guideline objectives, the diseases covered and the target population are well defined. The high-cost diseases guidelines showed higher scores (86% or 6.2) when compared to those of poverty-related diseases (74% or 5.4), although the latter still presented high scores.

It should be noted that the standard format of the HCDs accurately define the conditions covered by the guideline, using the codes of the International Statistical Classification of Diseases and Related Health Problems (ICD-10) and clearly describing the "inclusion" and "exclusion" criteria. Molino et al. (2016) and Ronsoni et al. (2015) also found higher scores in this domain, although their studies were restricted to assessments of clinical protocols for non-communicable diseases [9,12].

### Domain 2—stakeholder involvement

It evaluates the composition and degree of expertise of the group involved in drafting the guideline. In this domain, the ratings and quality scores dropped in both guideline groups; however the HCD mean scores (43% or 3.6) kept higher than those of PRD (26% or 2.6). The analysis revealed two weaknesses, namely the lack of commitment of all professional groups involved and the failure to promote participation of the target population. For instance, the sickle cell guideline (PRD 08), which had the lowest quality score in the domain (7%), was drawn up by only two medical specialists, with no reported participation of other health professionals or methodologists committed to evidence search and analysis.

An important difference between the assessed groups is that all the HCD guidelines were previously approved by the CONITEC and made available for public consultation and social collaboration in general. Only one guideline (PRD 14) of the PRD group was previously reviewed by the CONITEC and underwent a public consultation. None of the 32 guidelines evaluated in this study reported any kind of active patient participation in their development.

Effective patient participation in clinical guidelines development has been shown as a global difficulty due to issues concerning the closed structure of the Health Technology Assessment (HTA) area and patients' suspicion that the guideline developers might be influenced by pharmaceutical companies [15,16]. However, various studies have demonstrated that this strategy is crucial to improve the quality of the recommendations, since it allows identifying discordant points between professionals and patients, identifying priority needs from the perspective of users; points that are not well observed by guideline developers and managers which can help improve the strategies for guideline adherence and implementation [17,18].

### Domain 3—rigor of development

This domain showed one of the greatest disparities among the groups evaluated, where the quality score of the HCD guidelines, i.e. the compliance level with the criteria of the instrument, was almost seven times greater (48%) than that of the PRD guidelines (7%), and their mean score (3.9) was almost three times higher (1.4), respectively.

It was found that some key questions for guideline quality assessment were totally neglected in the PRD guidelines analyzed, for instance, description of the search methods, evidence selection criteria, strengths and limitations of the evidence, formulation of recommendations, consideration of benefits and harms, in addition to external review methodology and procedure for updating the guideline.

Among the guidelines evaluated, the Guide to Infectious and Parasitic Diseases (PRD 02) had the lowest quality score (1%). It is a publication intended to be a quick guide to basic care. However, it does not have a section informing the reader about the methods used to formulate the recommendations and it does not even provide bibliographic references. Notwithstanding, the Guide has the highest circulation among the publications evaluated, with 90 thousand printed copies.

Molino et al. (2016) and Ronsoni et al. (2013) reviewed some HCD guidelines of the Brazilian Ministry of Health and found similar quality scores in this domain, with mean values of 41% and 36%, respectively [12, 19].

Decrees No. 7508/11 and No. 7646/11 establish that the PCDTs of the Brazilian Ministry of Health must be updated every two years, conferring to the CONITEC the constitution or modification of such guidelines. All of the HCD documents reviewed in this study met these standards. Of the 16 PRD guidelines evaluated, two (PRD 02 and PRD 09) were published before those decrees and, among the others, only one (PRD 14) was published in accordance with the aforementioned decrees [4, 20, 21].

### Domain 4—clarity of presentation

This is the second domain with the highest scores, even with the trend of higher mean scores of the HCD guidelines (70% and 5.2) when compared to those of the PRD guidelines (61% and 4.6). In general, the guidelines in both groups were written in plain language, often addressing various aspects of the health issue.

The HCD guidelines are more concise than the PRD ones. The latter have a wide variety of formats and sizes of documents, contrasting with the first group, which had a standard format previously established by a specific ordinance that offered a roadmap for the PCDTs development, and consequently showed a more coherent presentation [22, 23].

### Domain 5—applicability

The “applicability” domain scored low in both groups. This domain includes questions that describe facilitators and barriers to the guidelines’ application, potential resource implications and monitoring criteria [11].

Despite the small margin of difference, the PRD guidelines scores were higher (33% and 2.9%) than those of the HCD guidelines (25% and 2.5%). This demonstrates a certain concern of guideline development panels with the application of the guidelines in contexts of greater operational difficulty. The normative character and little approach to guideline implementation actions were previously described by other authors who evaluated some PCDTs of the Brazilian Ministry of Health, finding results similar to those of the present study, such as those by Ronsoni et al. (2015), Ronsoni (2013) (37.5% and 3.3) and Molino et al. (2016) (34%) [9, 12, 19].

### Domain 6 –editorial independence

Similar to the "rigour of development" domain, this was one of the domains that showed the highest discrepancy between the scores of the HCD guidelines (43% and 3.6) and the PRD guidelines (1% and 1.1). This item evaluates the degree of reliability and equity in the guideline development. In this item, it is expected that there will be information and statements that demonstrate that the views or interests of the funding body did not influence the final recommendations.

The HCD guidelines did not reach higher scores solely because they did not specify more clearly the procedures used to avoid the funding bodies’ influence on their content and how potential competing interests were managed. However, there were supplementary documents and terms defining the procedures and responsibilities of each group of participants, and specifying that a declaration of competing interests was signed by all parties [12, 23].

On the other hand, the PRD guidelines did not have any similar procedure or standard, and there was no record attesting that a competing interest statement was signed or any actions were taken to avoid the funding bodies’ influence on the content and formulation of recommendations.

This points to a variability in the areas of the Ministry of Health concerning the procedures for declaring competing interests and how these conflicts are handled (or not). These are essential steps to ensure that the content of the recommendations is unbiased and the publications are reliable [2,3].

### International comparison

**Fig 1:**
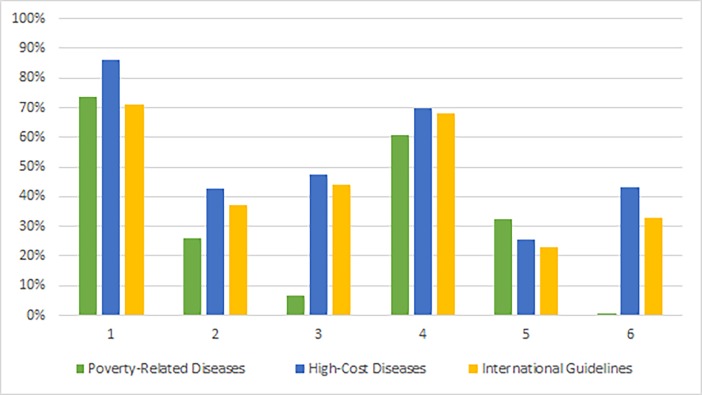
Quality scores of the AGREE II domains compared to the international guidelines in Alonso-Coello et al. (2010).

With the intention of analyzing the results from an international standpoint, the quality scores per domain were compared with the findings of Alonso-Coello et al. (2010), who carried out a systematic review of studies from around the world that brought together 625 different guidelines. Fig 1 was prepared with the most current data from that study, including guidelines published since 2003 [13].

It can be seen that, in general, the HCD guidelines are in line with international standards, with similar and slightly higher scores in all the domains evaluated.

The PRD guidelines, on the other hand, showed lower scores in most domains. The main contrasts can be noticed in the "rigor of development" and "editorial independence" domains, reinforcing the findings that these documents have methodological criteria problems in what concerns evidence-based health and the procedures adopted to guarantee an unbiased process.

### Overall guideline assessment

The comparison of this study’s results with international findings demonstrates the effectiveness of the method used. However, the participating appraisers did not consider it appropriate to individually assess whether a particular guideline would be recommended or not for use, as described in the AGREE II. Therefore, the appraisers decided to adopt criteria based on the analysis of previous domains.

Due to the greater emphasis on the "rigor of development" domain in similar studies [12,13,24,25], it was decided that this domain should be the central item for guideline assessment because of its relevance and greater number of analysis parameters.

According to the abovementioned criteria, the PCDTs for Rheumatoid Arthritis (HCD 17), Hepatitis C Virus Infection (HCD 18) and Breast Cancer (HCD 19) were considered as "recommended", and the other HCD guidelines (20 to 32) were considered as "recommended, with modifications". This result is in line with three other similar studies which pointed out that the PCDTs of the Ministry of Health, even with a methodological standard comparable to other international guidelines and superior to other Brazilian guidelines, need continuous improvement [9,12,19].

All the PRD guidelines (01 to 16) were considered as "not recommended" since, according to the AGREE II assessment, they did not meet the minimum criteria of the scientific method description, failing to provide whether or not the document was drawn up from an evidence-based health perspective.

The clinical guidelines with the highest score percentage in the AGREE II concern diseases that demand a heavy medicine expenditure of the Ministry of Health, namely, rheumatoid arthritis, hepatitis C virus infection and breast cancer, precisely those with a billion dollar budgetary impact for the Brazilian health system and for most health systems in developed countries [26, 27]. The lowest scores were of documents addressing the treatment of poverty-related diseases of low visibility such as intestinal parasitosis, human brucellosis, onchocerciasis, trachoma, among others. This indicates that the safety of patients suffering from such diseases and undergoing treatment cannot be ensured by the current treatment protocols.

This contrasts with the influence that budget pressure exerts on action priorities and investments in health qualification. This does not seem to be a coherent decision, since it is poverty-related diseases that have less potential for evidence generation due to less investment in clinical research and new technology development. Despite the decline in the number of cases of these diseases over the years, given the ongoing epidemiological transition, they still have a major impact on morbidity and mortality when we look at disease burden data, in addition to the contemporary risk of re-emergence of some previously controlled epidemics [6]. In addition, there is a trend for such issues to be less addressed in health training curricula, which consequently generates a greater need for consistent clinical guidelines [28].

## Final remarks

Based on the findings of this study, it is possible to verify a double quality standard in clinical guideline development of the Brazilian Ministry of Health, where the guidelines for global diseases that demand high-cost technologies showed higher scores in the AGREE II assessment when compared to those for poverty-related diseases which, normally, demand old and low-cost technologies.

Discrepancies found in quality and methods of selection, use of scientific evidence, and editorial transparency criteria are of concern and should be reviewed for the poverty diseases guidelines. Future studies must include the clinical credibility and implementation of guideline recommendations, as recommended by the new AGREE-REX [29]. This study did not include some of the most ordinary diseases, as those most frequent in primary health care services (such as diabetes and hypertension) and can add a broader view of guidelines development in Brazilian health system.

It can be inferred that the budgetary power and resource pressure also influence the action priorities and the quality of care in the public sector, making diseases that are neglected by the pharmaceutical market also be neglected at certain levels by the health system.

The neglect of the poverty-related diseases observed in this study concerns not only financial, scientific and product development investments but also the standardization and quality of the clinical services offered to the affected population.

## Supporting information

S1 File Links to guidelines. Links to clinical guidelines used in this study.
(DOCX)
