# Supplementary material for: The different clinical guideline standards in Brazil: High cost treatment diseases versus poverty-related diseases
Source: PLoS One. 2018 Oct 17;13(10):e0204723. doi: 10.1371/journal.pone.0204723 (PMC6192575; doi:10.1371/journal.pone.0204723)
Supplement: S1 File — Links to clinical guidelines used in this study. (DOCX) [file pone.0204723.s001.docx]

Dear Editors

All the data we have used in the study are completely available in the Brazilian Ministry of Health official website (32 clinical protocols), as described below:

<http://portalarquivos2.saude.gov.br/images/pdf/2015/fevereiro/06/guia-vigilancia-saude-atualizado-05-02-15.pdf>

<http://portalarquivos2.saude.gov.br/images/pdf/2014/janeiro/23/doen-infecciosas-guia-bolso-8ed.pdf>

<http://189.28.128.100/dab/docs/portaldab/publicacoes/guia_consulta_beriberi.pdf>

<http://189.28.128.100/dab/docs/portaldab/publicacoes/guia_evidencias_nutrisus.pdf>

<http://bvsms.saude.gov.br/bvs/publicacoes/manual_condutas_suplementacao_vitamina_a.pdf>

<http://bvsms.saude.gov.br/bvs/publicacoes/manual_suplementacao_ferro_condutas_gerais.pdf>

<http://portalarquivos2.saude.gov.br/images/pdf/2016/janeiro/14/dengue-manejo-adulto-crianca-5d.pdf>

<http://bvsms.saude.gov.br/bvs/publicacoes/doenca_falciforme_condutas_basicas.pdf>

<http://bvsms.saude.gov.br/bvs/publicacoes/vigilancia_esquistossome_mansoni_diretrizes_tecnicas.pdf>

<http://portalarquivos2.saude.gov.br/images/pdf/2016/marco/29/Protocolo-SAS-versao-3.pdf>

<http://bvsms.saude.gov.br/bvs/publicacoes/guia_vigilancia_filariose_linfatica.pdf>

<http://portalarquivos2.saude.gov.br/images/pdf/2016/fevereiro/10/Manual-t--cnico-operacional-diretrizes-eliminacao-hanseniase-4fev16-web.pdf>

<http://portalarquivos2.saude.gov.br/images/pdf/2015/janeiro/16/Leptospirose-diagnostico-manejo-clinico.pdf>

<http://conitec.gov.br/images/Consultas/Relatorios/2015/Relatorio_PCDT_IST_CP.pdf> <http://portalarquivos2.saude.gov.br/images/pdf/2014/dezembro/01/Manual-de-vigil--ncia-do-tracoma-e-sua-elimina----o-como-causa-de-cegueira.pdf>

<http://bvsms.saude.gov.br/bvs/publicacoes/prevencao_agravo_violencia_sexual_mulheres_3ed.pdf>

<http://conitec.gov.br/images/Protocolos/pcdt_ArtriteReumatoide_2015.pdf>

<http://conitec.gov.br/images/Protocolos/PCDT_HepatiteC.pdf>

<http://conitec.gov.br/images/Protocolos/DiretrizesDiagnosticasTerapeuticas_CarcinomaMama.pdf>

<http://conitec.gov.br/images/Protocolos/PCDT_ArtriteReativa.pdf>

<http://conitec.gov.br/images/Protocolos/DDT/DDT_CancerCabecaPescoco_2015.pdf>

<http://conitec.gov.br/images/Protocolos/PCDT_DoencaCeliaca.pdf>

<http://conitec.gov.br/images/Protocolos/PCDT_ELA.pdf>

<http://conitec.gov.br/images/Protocolos/PCDT_Retificacao_EscleroseMultipla_06.07.2015.pdf>

<http://conitec.gov.br/images/Protocolos/pcdt_hiperprolactinemia_2015.pdf>

<http://conitec.gov.br/images/Protocolos/pcdt_hipotirodismo-congenito_2015.pdf>

<http://conitec.gov.br/images/Protocolos/pcdt_ictioses-hereditarias_2015.pdf>

<http://conitec.gov.br/images/Protocolos/pcdt_insuficiencia-adrenal-congenita_2015.pdf>

<http://conitec.gov.br/images/Protocolos/pcdt_miastenia-gravis_2015.pdf>

<http://conitec.gov.br/images/Protocolos/ddt_Mieloma-Multiplo.pdf>

<http://conitec.gov.br/images/Protocolos/pcdt_guilain-barre_2015.pdf>

<http://conitec.gov.br/images/Protocolos/pcdt_uveites-posteriores_2015.pdf>
